# How is the discourse of performance-based financing shaped at the global level? A poststructural analysis

**DOI:** 10.1186/s12992-018-0443-9

**Published:** 2019-01-15

**Authors:** Lara Gautier, Manuela De Allegri, Valéry Ridde

**Affiliations:** 1 Department of Social and Preventive Medicine, School of Public Health (ESPUM), University of Montreal, 7101, avenue du Parc, 3rd floor, Montreal, Quebec H3N 1X9 Canada; 2 Public Health Research Institute (IRSPUM), University of Montreal, 7101 avenue du Parc, 3rd floor, Montreal, Quebec H3N 1X9 Canada; 3 Centre d’Etudes en Sciences Sociales sur les Mondes Africains, Américains et Asiatiques (CESSMA), IRD (French Institute for Research on Sustainable Development), Université Sorbonne Paris Cité, Case courrier 7017, 75205 Paris, Cedex 13 France; 4 Heidelberg Institute of Global Health, Medical Faculty and University Hospital, Heidelberg University, Im Neuenheimer Feld 130.3, 69120 Heidelberg, Germany; 5 IRD (French Institute for Research on Sustainable Development), CEPED (IRD-Université Paris Descartes), Université Sorbonne Paris Cité, ERL INSERM SAGESUD, 45 rue des Saints-Pères, 75006 Paris, France

**Keywords:** Global discourse, Performance-based financing, Diffusion entrepreneurs, Poststructural analysis

## Abstract

**Background:**

Performance-based financing (PBF) in low- and middle-income settings has diffused at an unusually rapid pace. While many studies have looked at PBF implementation processes and effects, there is an empirical research gap investigating the ways PBF has diffused. Discursive processes are paramount elements of policy diffusion because they explain the origins of essential elements of the political debate on PBF. Using Bacchi’s poststructural approach that emphasises problem representations embedded in the discourse, the present study analyses the construction of the global discourse on PBF.

**Methods:**

A rich corpus of qualitative data (57 in-depth interviews and 10 observation notes) was collected. The transcribed material was coded using QDAMiner©. Codes were assembled to populate analytical categories informed by the framework on diffusion entrepeneurs and Bacchi’s poststructural approach.

**Results:**

Our results feature problem representations shaped and spread by PBF global diffusion entrepreneurs. We explain how these representations reflected diffusion entrepreneurs’ own belief systems and interests, and conflicted with those of non-diffusion entrepreneurs. This research also reveals the specific strategies global diffusion entrepreneurs engaged in to effectively diffuse PBF, through reflecting problem representations based on the discourse on PBF, and inducing certain forms of policy experimentation, emulation, and learning.

**Conclusions:**

Bacchi’s poststructural approach is useful to analyse the construction of global health problem representations and the strategies set by global diffusion entrepreneurs to spread these representations. Future research is needed to investigate the belief systems, motivations, resources, and strategies of actors that shape the construction of global health discourses.

**Electronic supplementary material:**

The online version of this article (10.1186/s12992-018-0443-9) contains supplementary material, which is available to authorized users.

## Background

The beginning of the millennium saw the rise of Universal Health Coverage (UHC) as an overarching objective shaping health sector reform in low- and middle-income countries (LMICs). Since then, strategies to increase coverage and access to quality health services have been (re) framed by international actors as being part of a global movement towards achieving UHC. Performance-based financing (PBF), a health systems reform that shifts from an input-based to an output-based purchasing approach, provides a classic example of continuous “reframing”. As of June 2017, no less than 32 out of 46 (71,7%) sub-Saharan African (SSA) countries utilised PBF [1].

If the claims in favour of developing PBF in Africa are to be gauged, it is critical to assess not only PBF implementation processes and effects [2–4], but also how it gained traction at a global, continental, and national level. To date, there has not been any empirical investigation on policy diffusion processes at the global level. PBF evolves in global governance characterised by increasing polycentrism, whereby international institutions (i.e., multilateral donors, bilateral donors, United Nations agencies, and non-governmental organisations (NGOs)), networks, and key individuals represent political units exerting power. At the global level, the discourses produced by actors of polycentric governance cannot be overlooked. Building on the notion of “diffusion entrepreneurs” (i.e., collective and individual actors actively promoting a global policy) and on our prior work having developed a framework specific to PBF diffusion entrepreneurs [1], we investigate the content (*what*) of their discourses and *how* these discourses matter for global diffusion.

We understand discourse as “a dynamic form of social practice which shapes the social world including identities, social relations and understandings of the world” [5]. The literature on the shaping of global health discourses is expanding [6–9], yet few empirical papers have addressed health financing strategies, and even fewer have used a framework encompassing polycentrism as a starting point for analysis. A well-known example is Lee & Goodman’s description of a powerful “global elite” made of a wide array of actors who backed the introduction of user fees in LMICs [10]. A recent book [11] analyses the discourse that transpires from the contents of a major PBF web platform, but this analysis only reflects the views of the people behind that specific platform. The deep-rooted processes that shape diffusion entrepreneurs’ representations of global health financing issues and how these representations specifically mould the global discourse on PBF remain to be unravelled.

In this paper, we examine the content of the discourse on PBF and how it is brought about in the global health arena, emphasising the contribution made by diffusion entrepreneurs acting at the global level.

## Methods

### Theoretical underpinnings

#### Diffusion entrepreneurs’ framework

In polycentric governance, various units of governing authorities (including networks and individuals at the lowest level of governance) influence global policymaking [12]. Diffusion entrepreneurs (DEs) represent those units of governing authorities acting to spur the diffusion of their favoured policies. DEs are shaped by representation systems that reflect their individual or collective culture. These ideational representations influence their career choices and ensure motivation to support a particular policy [13]. Once DEs acquire sufficient (financial, expert, social, etc.) resources and statutory authority on the global arena, their voice is bound to have a significant echo [1].

In our prior work, we identified PBF DEs [1]. At the global level, they include a wide range of individuals (academics, experts, consultants, and employees of international organisations), international organisations (i.e., NGOs, bilateral development agencies, and a multilateral development bank), and transnational networks particularly active in SSA. Also in our prior work, we proposed that in order to foster PBF diffusion worldwide, DEs deliberately engage in specific strategies to frame the PBF policy in certain ways, to stimulate policy emulation, to shape certain forms of policy learning, and to facilitate policy experimentation [1].

#### Bacchi’s poststructural approach

Several interpretivists (e.g., Kingdon [14], Rochefort & Cobb [15], etc.) and critical realists (e.g., Pawson & Tilley [16]) have been interested in the way(s) in which policies are defined and framed, often specifically investigating how the discourse on given policies is produced. There are two shortcomings to these approaches [17]. First, both lack a reflection on how contexts, subjects (e.g., targeted populations), and problems are conceptualised. For instance, in his discussion of problem definition and framing, Kingdon leaves ambiguous the contested nature of problems reaching the political agenda [14]. Second, critical realists and interpretivists neither question the representation systems reflected in problem definition nor the nature of knowledge produced (and/or used) to define and frame policies.

Poststructuralists (e.g., Bacchi) argue that social actors ought to be understood to be “in continual formation” and therefore “form part of what must be ‘interpreted’ rather than the starting point of interpretation” [17]. Bacchi’s conceptual framing, aka “*What’s the problem represented to be?”* (WPR), starts with a postulation and identifies the problem representation implicit within it. Bacchi’s approach is rooted in Foucaud-inspired concept of ‘governing’. She suggests that governing “takes place through the formation of ‘problems’, that is, through problematisation” [17]. Governing units not only incorporate classic political actors (government, political parties etc.), but also units at lower levels of policymaking—experts and professionals [17]. For Bacchi, these new governing units bring new questions to policy analysis: their discourse produces problems within the policy solutions they advocate for [18]. She suggests analysing the process of “problematisation” to reflect upon the overall shaping of policies, in order to identify what this process encompasses as well as what it overlooks (Table 1)*.* Bacchi describes WPR as a poststructural approach in the sense that “‘subjects,’ or ‘problems’ that form the basis of policy analysis are understood as shaped, or constituted, through practices” [17]. Bacchi does not entirely reject previous works: she simply suggests to dig deeper into the context surrounding definition and framing of policies (i.e., how representations affect this context, or how context affects representations). Bacchi’s framework incorporates a few dimensions (e.g., categories of problem definitions) that several interpretivists looked into in greater detail (e.g., [15]).

**Table 1:** Bacchi’s WPR approach (Adapted from Bacchi [68])

Question #	Question title	Explanation
WPR Q. #1	What’s the problem represented to be in a specific policy or policies?	If a government proposes to do something, what is it hoping to change? And, hence, what does it produce as the 'problem'? Here, considering policy ‘objects’ and ‘subjects’ (i.e., people who become problematised)
WPR Q. #2	What deep-seated presuppositions or assumptions underlie this representation of the “problem” (problem representation)?	Looking into representation systems embedded in the discourse
WPR Q. #3	How has this representation of the “problem” come about?	Analysing power relationships, the role of conflicting ideologies, disrupting the assumption that what *is* reflects what *has to be*
WPR Q. #4	What is left unproblematic in this problem representation? Where are the silences? Can the “problem” be conceptualised differently?	Identifying what has been overlooked and looking at the implications of these silences
WPR Q. #5	What effects (discursive, subjectification, lived) are produced by this representation of the “problem”?	Identifying the perceived effects of the problem representation
WPR Q. #6	How and where has this representation of the “problem” been produced, disseminated and defended? How has it been and/or how can it be disrupted and replaced?	Identifying the governing knowledges, sites, institutions, and networks involved in the problem representation

While we find the WPR approach helpful in our research on the global PBF discourses, we explicitly distinguish Bacchi’s and the criticism on which it builds of other policy analysts, from our conceptual framework on DEs. The starting assumption of this empirical study is that PBF discursively emerged as a policy innovation that encompassed certain problem representations. We propose that the problematisation process reflects the interference and influence of powerful actors – global DEs. DEs are governing units evolving in polycentric governance [17]: their nature and characteristics as much as their discourse [1] ought to be interpreted. Our approach includes (and interprets) global DEs as producers of problem representations in the global health arena.

### Analytical framework

Our results are charted using DEs’ framework dimensions, while the WPR approach provides analytical categories to critically reflect on and further unpack the results. Thus, Bacchi’s six exploratory questions are brought into diffusion entrepreneurs’ key characteristics and strategies (detailed in [1]) (Table 2). We also use Rochefort and Cobb’s ‘categories of problem definition claims’ (outlined below in Table 2), as they fall under the scope of Bacchi’s first question.

**Table 2:** Analytical framework (adapted to analyse the global discourse on PBF)

1. Describing the discourse on PBF as a policy *solution*	
Describing PBF policy representations, by comparing PBF definitions across four generic manuals [24–27] and one institutional position paper [23] developed by organisational diffusion entrepreneurs, and definitions provided by diffusion entrepreneurs in interviews.	
2. Describe WHAT is promoted, i.e. PBF *problem* representations	
DEs’ theoretical framework dimensions	Bacchi’s WPR questions
DEs’ representation systems and how they are reflected in PBF problem representations	*WPR Q. #1: What is the problem represented to be in the PBF policy?* • *Causality*: selectively identifying the causal patterns leading to the problem, including culpabilising those considered responsible • *Severity*: “how serious a problem and its consequences are taken to be” [15] • *Proximity*: characterising the issue in a way that appeals to personal experience/emotions or concerns a matter that feels close to home • *Problem populations*: characterising groups and individuals affected by the problem *WPR Q. #2: What deep-seated presuppositions or assumptions underlie this representation of the problem?* Describing how DEs’ representation systems, i.e. DEs’ personal, collective, and institutional cultures that are reflected in their assumptions about the world, shape these problem representations.
DEs’ motivations to deal with the problem; resources at hand (i.e., knowledge, financial, social, political and temporal resources), and capacity to demonstrate authority at the global level. Four types of authority are distinguished [1]: • *Financial authority* supposes a recognised status in the global arena mostly stemming from the large amounts of financial resources fuelled into international development cooperation • *Expert authority* may be achieved when entrepreneurs pursue an internationally-recognised status of expertise, mainly through mobilising knowledge, social, and temporal resources • *Scientific authority* involves both building international renown and putting forward the validity or utility of the claimant’s “definition, description or explanation of reality” [69] which secures a legitimate normative power • *Moral authority* stems from the status of the claimant *vis-à-vis* those whose behaviour they seek to shape, and from the validity of the categories that the claimant uses to express the needed political changes [70]	*WPR Q. #3: How has this representation of the problem come about?* Attempting to answer this question using empirical data on DEs’ motivations to fuel their problem representations, DEs’ resources at hand, and DEs’ types of authority.
N/A	*WPR Q. #4: What is left unproblematic in this problem representation?* Attempting to answer this question using empirical data: critically reflecting on DEs’ representations of what PBF is supposed to solve, and looking into the criticism expressed by several key informants towards DEs’ discourse
N/A	*WPR Q. #5: What effects (discursive, subjectification, lived) are produced by this representation of the problem?* Attempting to answer this question using empirical data, looking at DE’s representation of what PBF is supposed to solve, identifying the perceived discursive effects, and the “subjectification” (i.e. the making and unmaking “subjects” [68]) that is operated by DEs NB. We do not consider the “lived effects”, since the policy considered here can hardly bear an impact on life or death – at least not in the sense Bacchi conceives this analytical subcategory.
3. Analyse HOW PBF policy and problem representations are promoted by diffusion entrepreneurs	
How do DEs link PBF to common popular frames (*policy framing*), which in turn creates the conditions of successful pilot programmes (*policy experimentation*), appeals to a sense of community (*policy emulation*); and gets fuelled through multiple forms of knowledge (*policy learning*)?	*WPR Q. #6: How and where has this representation of the « problem » been produced, disseminated and defended?* Investigating how problem representations are defended by DEs

### Data collection

From November 2016 to November 2017, the first author collected data from informants (*N* = 57) through in-depth interviews via a snowball sampling approach, and from participant observation in PBF-related international workshops, webinars, and meetings (*N* = 10). Key informants primarily included DEs intervening at the global level that were identified through prior mapping [1], i.e. employees of international organisations and NGOs involved in PBF promotion; academics; international experts; and facilitators of PBF transnational networks. We also interviewed “non-DE” informants, i.e. people who are not proponents of PBF, yet were acknowledged as PBF experts at the global level (e.g., academics) and/or who have been directly interacting with DEs along the course of their career or as collaborators involved in PBF training, pilot scheme experimentation, and evaluation of PBF schemes (e.g., employees of international organisations, SSA policymakers, and SSA consultants involved in PBF experimentation). SSA informants were included to provide additional insights into the activities undertaken by global DEs. All interviewees were approached by email. The first participants were recruited on-site during a major global health systems event [19]. The first author carried out all interviews in English or French. Interview guides included 25 open-ended questions approaching various themes reflected in the DE framework (Additional file 1). Two questions prompted respondents to quote PBF reference documents and resource persons. All participants read a detailed information sheet and provided their written consent prior to the interview. Ethical approval was obtained from University of Montreal’s *Comité d'éthique de la recherche en santé*.

To increase confirmability, we carried out two triangulation exercises. First, to verify or complement material, we undertook additional interviews with seven key DE informants previously interviewed. Second, in August 2017, after verbatim transcriptions were completed (by the first author aided by three research assistants), we electronically sent all the participants a two-page description of preliminary results. Complements sent by participants in response to that email were subsequently added to the corresponding transcriptions. Saturation was achieved through recognising that no new data was emerging in the interviews [20].

Table 3 provides details of the 57 respondents. Among the 57 key informants, 35 were individual DEs (promoting PBF as individual entrepreneurs, speaking on their own) and/or operational employees of organisational DEs (i.e., transnational networks, NGOs, or international organisations). The majority of them were medical doctors (*N* = 20), public health (*N* = 15) or health economics (*N* = 2) specialists, and economists (*N* = 10).

**Table 3:** Participants’ general characteristics

Current affiliation (*N* = 57)		Main educational background (*N* = 57)		Years of experience in international development, all but “NATGOV” cat. (*N* = 44)	
International organisation [INTORG]	19	Medical sciences	33	< 10 years	6
National Government (SSA countries) [NATGOV]	13	Economics	15	> 10 years < 20 years	27
Independent consultant [INDCONS]	10	Other social sciences	4	> 20 years	11
Academic Institution [ACADINST]	7	Other health sciences	4		
Private for profit [PRIVFP]	4	Gender (*N* = 57)			
Private non-for-profit [PRIVNFP]	3	Male	45		
Other [OTHER]	1	Female	12		

### Data analysis

Using a primarily deductive approach, the material was coded by the first author using QDAMiner©. Codes were assembled to populate analytical categories, which consisted of the abovementioned dimensions integrating the DE framework and Bacchi’s six WPR questions. We allowed additional subdimensions to emerge from the data in an inductive fashion. Based on five interview transcripts, a preliminary codebook was shared with co-authors of this paper and subsequently adjusted before continuing the coding process [21]. All analytical thoughts were brought into QDAMiner© via the memo feature, and were used to further reflect on the data analysis [22].

## Results

Findings are presented as follows: first, we report on the ways the global definitions of PBF reflect DEs’ representation systems; second, we show how the problematisation of PBF brings about DEs’ motivations, resources, and authority; and third, we describe the strategies developed by DEs to promote these problem representations.

### How DEs’ representation systems are reflected in the definition of performance-based financing (PBF) as a policy solution

Informants quoted five key reference documents (including four generic manuals and one PBF position paper) on PBF [23–27], which also happened to be developed by organisational DEs. We interviewed at least one representative of each of these organisational DEs. We extracted the definitions used in these five documents (Table 4).

**Table 4:** PBF definitions contained in reference documents and corresponding language categories

*Source*	*Definition*	*Main keywords and their language categories*			
The World Bank’s Performance-based financing Toolkit (2013) [24]	“PBF targets health facilities with a fee-for-service (conditional on quality) payment mechanism. […] PBF involves contracts with individual health facilities, whether public or private […]. PBF is done through a ‘contracting-in’ approach: PBF is put onto existing public and private health systems with a significant involvement of nonstarter actors”.	*Economic sciences language*	*Management sciences language*	*Clinical language*	*Social sciences & humanities language*
		*Conditionality (incentive theory)		*Quality of care	*Health systems reform
		*Contract (contract theory)			
SinaHealth coursebook (2017) [25]	“Performance-based financing is a systems reform approach, which offers an answer to the 'how' of achieving Universal Health Coverage and the Sustainable Development Goals 2015-2030. Unlike other financing mechanisms, PBF proposes a hierarchy whereby the delivery of quality services comes first, followed by the efficient use of scarce public resources and only then equity and financial access”.	*Service delivery		*Quality of care	*Systems reform
		*Efficiency			*Equity and financial access
		*Financing mechanism			
PBF Handbook by Management Sciences for Health (MSH) and USAID (2011) [27]	“PBF is the transfer of money or material goods from a funder or other supporter to a recipient, conditional on the recipient taking a measurable action or achieving a predetermined performance target. […] PBF shifts most financial risk from the funder to the recipient: payment (or sometimes the ‘performance incentive’ portion of the payment) is received when—or withheld until—results or actions are verified by the funder. […] [T]he funder links incentives to the recipient’s achievement of predetermined results. Recipients include institutions and/or individuals; in a health program, supply-side recipients might be service-providing institutions (clinic, hospital) and/or health care providers at any level”.	*Conditionality (incentive theory)			
		*Money transfer			
		*Incentives			
		*Service delivery			
		*Measurable action/target			
Royal Institute of Tropical Medicine (KIT) booklet (2011) [27]	“We use ‘performance’ in terms of productivity (number of outputs, rather than attaining targets or coverage of certain priority programmes) and of quality of care as perceived by the patient as well as by professionals. […] RBF, PBF, P4P or ‘achat de performance’ all aim at motivating healthcare workers to perform better. To achieve this, one can stimulate both their intrinsic motivators […], as well as their extrinsic motivators such as financial incentives”.	*Production of healthcare	*Outputs	*Quality of care	
		*Incentives			
		*Motivation			
Cordaid position paper (2015) [23]	“Results Based Financing [RBF] is a system strengthening approach that introduces checks and balances along the service delivery chain, encouraging better governance, transparence and enhanced accountability. It achieves this by linking payments directly to performance. Contrary to traditional input funding, service providers […] receive their payment on the basis of agreed indicators and verified output. […] They are autonomous in how they spend the funds in order to achieve their own aims […]. RBF motivates service providers to deliver more services of higher quality and promotes entrepreneurship”.	*Conditionality (incentive theory)	*Governance, transparence and accountability	*Quality of care	*System strengthening approach
		*Measurable action/target			
			*Autonomy		
			*Entrepreneur-ship		
			*Verification of outputs (output evaluation)		
		*Motivation			
		*Service delivery			

NB: Some DEs, such as Cordaid, used the expression “results-based financing (RBF)” for what is generally referred to as performance-based financing (PBF). Usually, PBF is encompassed in RBF, as it represents a supply-side type of RBF [71]. Other DEs use the expression “pay[ing] for performance (P4P)”

Table 4 highlights the focus on the supply-side of the health financing equation. It illustrates the high prevalence of economic language in PBF definitions used in reference documentation produced by organisational DEs, and referred to by DE informants. This depiction is consistent with DEs’ discourse when they described PBF during interviews:

*It came about as an innovative financing, right? I mean, a lot of inputs financing has been done, and continues to be done, and the thinking is that… motivation and focusing on results, might bring about change… and the performance of the system. And… the tools […] are… Tools that could… make the system more effective and efficient. And dynamise how people look at health services and delivery of services.* (I53_INTORG)

DEs defined PBF using an *instrumental orientation*, by emphasising a logical course of action to achieve outcomes or “results”, rather than the means of implementation [15]. Informants described this feature as a major innovation, since input-based financing was, by contrast, directed primarily to means of implementation (e.g., training). According to DEs, PBF emerged as an innovative policy solution that offered opportunities for creativity and transformative practices in health facilities, thanks to enhanced motivation and autonomy. Thus, PBF was initially brought up with much enthusiasm, sometimes described as a “*magic bullet*” (I02_INTORG) that would solve a wide range of health system issues (see Methods).

The economic wording highlighted in Table 4 reflects the idea that DEs shared the same economic language. Both DE and non-DE respondents referred to DEs’ training background in (health) economics, which spurred an early interest in applying economic concepts to health systems. Such background would lead them to be “*in complete agreement with the logic of tools and approaches*” brought by PBF (I23a_ INDCONS). Thus, notions embedded in PBF such as separation of functions and contracting, which borrowed from economic theories (i.e., contract, incentive, and principal-agent theories), were comprehensible to DEs trained in economics. However, we noted some emerging differences of ideological positioning between informants who acted as DEs: some favoured institutional arrangements, while others preferred private sector principles (e.g., PBF enabling an increased competition between providers). Thus DEs bought into different economic cultures, and did not necessarily agree on the specifics of how to yield the best possible efficiency. As for organisational DEs, an institutional culture rooted in economic tradition appears to have shaped their worldview:

*R: The World Bank was very interested. I think… the market thinking, fitted… with their ideology.* (I04_PRIVNFP)

Such economic background shaped DEs’ assumptions about the world. According to non-DE respondents, economists are, by nature, willing to quantify complex phenomena: PBF precisely matches this representation as it prominently features quantitative indicators and payment-by-result. Yet for non-DEs, it is problematic to have performance measured by single indicators that are supposed to capture performance on complex health system process, such as those related to HIV or maternal care. Non-DEs also suggested that economists’ representations of healthcare are driven by a tendency to set a price on anything deemed valuable. For non-DEs, this tendency involves philosophical challenges as much as practical challenges: can one claim, “*being able to objectively transform [health issues] into numbers*”? (I34_ACADINST).

Besides economics, many interviewees mentioned a PBF anchor in management sciences, and in particular in relation to new public management (NPM). Indeed PBF entails a stronger accountability at all levels of health system management. DE respondents suggested that PBF was conceived as a way to critically curb corruption in African countries. This worldview emphasising accountability and transparency was described in contrast with traditional ways of managing development efforts.

The clinical discourse, apart from quality of care (which does come as central in DEs’ language), was less prominent. Several non-DE clinicians criticised PBF for being unsustainable and creating inequitable access to healthcare, because health workers would be more likely to work where they can earn more.

Other non-DE respondents used the above-mentioned arguments to criticise PBF – lack of sustainability and risks of unintended consequences for health equity. The debate on health workers’ motivation, mostly brought up by non-DEs, was a case in point. For DEs, improving staff pay through financial incentives would lead to better retention of health workers. Non-DEs voiced concerns about PBF being a piecemeal reform that was just about “*paying [health workers] through incentives*” (I05_INTORG), and offered supportive supervision and/or salaries of health workers’ increase as alternative policy solutions (I31_ACADINST). This criticism prompted DEs to shift the discourse. DEs thus emphasised the fact that PBF could close the *can do-will do* gap, not only through provision of financial incentives, but also by increasing resource generation to enable better performance through work environment improvements and closer performance feedback cycles. In general, the debate between “pro-PBF” and “anti-PBF” communities led DEs to reframe PBF as being more comprehensive than pay-for-performance. DEs crafted PBF as system-oriented reform, which could serve as a “*cloth-hanger*” to leverage other reforms (I16b_ INTORG).

### How are DEs’ characteristics reflected in the problematisation of PBF?

#### What are the problems represented to be in performance-based financing?

DEs highlighted a number of health systems problems in LMICs that PBF intends to solve. Table 5 outlines DEs’ representations of the problems as they appear in key documentation produced organisational DEs.

**Table 5:** DEs’ representation of the problems (based on definitions extracted from [23–27])

Represented problems that PBF intends to solve	Related quotes
1. Input-based financing systems with passive strategic function causing public service ineffectiveness and inefficiency)	*Let’s no longer speak about how many health facilities are being built, how many staffs are being trained […]. Because [ministries of finance in LMICs] have been putting a lot of money into... into input-based financing for a long time and not necessarily seen results thereafter.* (I03_INTORG)
2. Lack of accountability of public health spending	*You cannot ask somebody to manage something like… two billions, three billions as incomes… to put that in a system; and believe that he will do that… properly… No! He must live… And… He injects the 2 billions… without earning anything from those 2 billions. Well: I do not think that in… in-in-in other… in any other country this can work out, when you know that he gets paid 200,000 francs. What do… What do you expect? (laughs). While if… based on his efficacy to inject the two billions in the health system, there was something… formal, clear… [that enabled him to keep some money for himself] I am sure he would… he would be eager to do his job properly.* (I49_NATGOV)
3. Unmotivated and underperforming health workers	*In most African countries, people... people are not... well paid. I think that... with the salaries that people... the remuneration must get to a fair value. And... in... in this [PBF] system, it's well-known: if you work better, you’ll get more bonuses. So your work is recognised in value.* (I41_NATGOV)
4. Highly centralised decision-making (i.e., for health planning and management)	*A big problem in [African] countries that I’ve seen… is that they are all centralised: you want something, you have to go to the ministry of health, and talk to the director… of procurement, et cetera… to get a status quo. For me, I think it's scandalous; we must stop, we must completely change.* (I33_INTORG)
5. Underperforming monitoring systems	*I think information and transparency is something that’s extremely important. Hum… Again, taking a context like [country name removed] where there is no health information system, it just doesn’t exist: they’ve tried a thousand times, through a dozen of different ways, and it just never gets up and running.* (I08_INTORG)

These problems often directly appealed to a strong sensation of *proximity* relating to DEs’ own personal experience:

*So I was working at the ministry [of Health]. […] I knew very well that… we had a lot of losses, we were buying communication equipment, printing a lot of things… and […] this was getting… dusty, so money was being wasted.* (I54_INDCONS)

For some employees of organisational DEs, the sense of proximity also owed to the wave of market-oriented reforms in high-income countries that aimed at cost containment, including pay-for-performance reforms. This movement, being high on the agenda in several European countries, was embraced by several non-profit organisations working in LMICs. However, the sense of *severity* was particularly linked to African contexts, where the under-utilisation of healthcare services and the suboptimal quality of healthcare service delivery were described as salient.

These representations of health systems problems related to a number of selective *causal* patterns, which mostly featured economic frames and, in particular, related to efficiency and governance. For DE respondents, the lack of decentralisation (of decision-making) was considered the main cause for absenteeism and suboptimal service delivery in general. DEs suggested that centralised decision-making in African countries was obfuscating the swift transfer of financial resources to health facilities.

At peripheral level, and in facilities in particular, centralisation was perceived (by both DEs and non-DEs) as a demotivating factor as it removed the ability to make independent decisions. In addition, informants frequently mentioned inadequate work environment as a key determinant of low motivation. For DEs, healthcare human resources’ critically lacked financial incentives to do their job adequately. According to DE respondents, the combination of poor salaries and working conditions drives low motivation, leading to inefficiency and poor governance. Poor governance was illustrated by suboptimal data reporting (e.g., inaccurate patients’ records). Participants expressed the need to break away from this form of “*business as usual*” (I03_INTORG). Many interviewees suggested that health workers should be held accountable towards the health system; while others mentioned the need to be accountable toward African populations. Two *problem populations* emerge from this discourse: first, African people, who suffer from being underserved and getting poor services; and second, health workers, who may be represented either as executioners or victims of an inefficient system that underpays them.

#### What deep-seated presuppositions or assumptions underlie these representations of the problems?

As said above, DEs share the same broad values owing to economic-oriented assumptions, which shaped their world representations. However, individual DEs’ personal history specifically shaped their problem representations. For instance, a respondent cited his experience in Mozambique, at the time of the Marxist-Leninist regime, where, in opposition to that model he “*realised the importance of market mechanisms*” (I10a_PRIVFP). Several individual DEs were cognisant of this shaping process, willingly admitting that their own assumptions of the world, mainly driven by their personal history, shaped their inclination to promoting PBF. Their history influenced their solution-oriented framing of public health issues:

*So I'm pretty transparent: I'll say, I'm a political entrepreneur, I want to have an impact, and so I work on solutions. That's the first thing: I do not see myself as working on problems, […] I like to have an application, telling myself: it can have an impact, it can help solve a problem.* (I19a_ACADINST)

Several individual DEs could explicitly recall how they sought economic degrees to make sense of their personal experiences, enabling them to tie their policy solution (PBF) to economic concepts.

#### How have these representations of the problems come about?

First, the perception that “nothing else works”, which built on DEs’ assessment that these problems of health service ineffectiveness and inefficiency, and low utilisation of healthcare services in general, were not adequately dealt with through the existing policies (e.g., health insurance), is likely to have won the global arena’s opinion. This widespread perception led several big global health players to look for an effective catalyst for change, which PBF was – at least on paper – bringing about. Non-DEs described PBF as a policy solution that rapidly spread on a deserted, yet fertile, field owing to the “*paucity of public health innovations*” and the relative failure of existing policies (I34_ACADINST).

Second, DEs’ problem representations gained traction because DEs benefited from a worldwide reputation built on recognised skillset and authority: their discourse was deemed credible. All DE informants had 10 years or more experience in international development when they started working on PBF. Their professional seniority enabled them to develop critical knowledge, social, and political resources. Such resources also secured easy access to political leaders. These resources, combined with an expert and/or scientific authority, provided individual DEs with credibility on the global arena. Organisational DEs were perceived as possessing strong moral, expert, and financial authority, making them *“recognised and respected international agencies”* (I31_ ACADINST).

When individual DEs lacked a type of resource (e.g., temporal resources), informants asserted that they could tap into other resources categories (e.g., knowledge resources) to enhance their credibility and thus widen their influence. In addition, many DEs (including our interviewees) personally knew each other. Some worked side-by-side in various settings, most often on the occasion of PBF (or performance contracting) experimentation in Haiti, Afghanistan, Cambodia, and later on in SSA countries. In the beginning of the 2010s, joining all types of resources, a “*network of PBF experts and consultants*” (I04_PRIVNFP) regularly met. Connections between respondents appeared clearly: DE informants could name at least two individual DEs from outside their organisation, and also mentioned an organisational DE distinct from their own organisation. Many respondents (N=30) also claimed being members of a transnational network acting as DE. Due to close affiliations, DEs’ viewpoints about the inefficiencies and poor governance they perceived as hampering the health system could be easily spread.

Third, informants referred to a general trend towards increasing efficiency at the global level, due to perceived insufficient gains in health. Hence the ‘output-based aid’ trend became popular among donors. PBF was seen as an opportunity for them to (finally) spend money that served “*to buy deliveries and vaccinations, antenatal care visits, et cetera”* (I02_INTORG). The 2004 World Bank Report on service delivery was also mentioned as playing an instrumental role in setting the stage for such representations of the problem to emerge. The report’s emphasis on service delivery was directly linked to the reflection about LMIC public systems’ accountability.

Hence, the discourses on efficiency and on the possibility to enhance efficiency through improved purchasing came about. These discourses found an echo in organisational and individual DEs who were depending on donor funding. DEs expressed strong assumptions about the legitimacy of the results-based language. Based on the powerful “value for money” token, a DE informant said that, “*any institution would be sensitive to this language”.* (I36_PRIVFP). Indeed, these economic-driven representations provided them the opportunity to reinstate their position as global health leaders:

*At a certain point 10 years ago… […] Their [the World Bank’s] own projects were so… hopeless, in a way, and so disappointing […], that when they discovered PBF… […] they realised: well this may be a very good thing to regain initiative in the public health… sphere.* (I10a_PRIVFP)

Promoting a policy that carried these economic-driven representations was expected to serve DEs’ interests by enhancing their visibility in a global health systems arena increasingly leaning towards better efficiency. Employees of organisational DEs also had “*incentives to get in that business*” given that PBF had a “*high visibility*” inside their organisations and that they could “*get extra money to implement the [PBF] project and make the whole health project more successful*” (I16a_INTORG). Several individual DEs were aware of the need to position oneself within the global health arena. For several interviewees, DEs had genuine motivations to solve public problems, so that they could say that they had “*brought something important for the system*” (I34_ACADINST).

#### What is left unproblematic in these problems representations?

Both DEs and non-DEs identified areas left unproblematic in PBF problem representations. For non-DEs, economic- and managerial-oriented representations left out key issues that mobilise other types of worldviews, which relate to systemic approaches. First, there was a perceived lack of consideration for broader systems reforms. Non-DEs suggested that by introducing PBF as a “magic bullet”, policy reform might draw attention to secondary issues, such as provider incentives, instead of addressing more fundamental problems, such as the need to reform public service in LMICs. An interviewee (I18_INTORG) metaphorically spoke of DEs’ promoting “*icing on the cake with no cake*”, with healthcare services not yet structured to be able to integrate such reform. Other – less critical – informants (including several employees of organisational DEs) perceived PBF as an instrument that needed to be complemented with more systemic reforms, such as social health insurance. On the contrary, individual DE respondents (and at least one DE reference document [26]) tended to present PBF as the primary system to put in place before engaging in additional reforms.

Second, non-DEs feared that by framing the issues of suboptimal quality and use of healthcare services exclusively from the supply-side perspective, PBF promoters would overlook population’s demand for quality healthcare. For instance, equity concerns were only raised by four DE informants, and among those, one of them asserted that equity should only come after “*mak [ing] sure there are enough resources in the health centers*” for healthcare service delivery (I10b_PRIVFP). Some DEs portrayed non-DEs – at least those prioritising equity – as “idealists”, as they were eluding the major issue of availability of resources.

#### What effects (discursive, subjectification) are produced by these representations of the problems?

For Bacchi, “some problem representations create difficulties for members of social groups more so than for members of other groups” [28], i.e. they produce discursive effects that could be potentially harmful. Non-DEs emphasised how health system ineffectiveness –postulated by DEs – combined with overlooking systemic issues had critical implications for populations. Furthermore, non-DE respondents pointed to risks of echoing these discourses in an environment where basic structures and resources are lacking. These informants could often substantiate allegations of perverse effects with concrete examples, such as health workers’ gaming observed in several countries experimenting PBF.

Problem representations also produce subjectification of those who are considered agents of the problems. In the case of PBF, these are health workers and bureaucrats working under centralised input-based financing systems. Subjectification of health workers was identified in problem representations conveyed by both DEs and non-DEs. DEs produced a discourse of “responsibilised subjects” able to develop a sense of entrepreneurship to ensure quality service delivery. Coherent with Bacchi’s assessment, health workers would not automatically develop entrepreneurial subjectivities. However, the continual linking of healthcare practice to a simple managerial activity whereby health workers are supposed to attract patients, reflected DEs’ intention to induce such “paradigm shift”. Non-DE respondents argued that the strong (individual) accountability that was attached to these subjects might have diverted attention from more structural factors that critically shape the delivery of healthcare services. Besides, such subjectification could be frustrating for health workers evolving in complex environments because of a limited control over other parameters. For DE informants, such discourse was counter-productive because it tended to convey an image of passive health workers.

For DE informants, the key to improve salary and working conditions was to introduce incentives. Non-DEs often criticised this form of financial motivation: for them, developing this form of extrinsic motivation was reducing health workers to venal subjects. In turn, DEs portrayed non-DEs as overlooking the contexts where health workers were evolving, i.e. earning very little. In fact, non-DE respondents also voiced a number of discourses about health workers that conveyed strong values about human motivation, whereby altruistism would be opposed to financial gain-orientated behaviours:

*Basically, there's intrinsic motivation: it’s good, it’s altruistic, it’s ‘cool’… And extrinsic motivation: it’s bad… […] Well, I find this is an ultra-binary vision of how an individual works!* (I20a_INTORG)

In sum, both DEs and non-DEs produced simplistic discourses that tended to reduce these subjects to binary categories. It often seemed that both groups were building their discourse (and, in some ways, their identity) in opposition to the problem representations conveyed by the other group.

### How do diffusion entrepreneurs promote PBF?

Diffusion entrepreneurs engaged in an apparatus of strategies to foster the production and diffusion of their representations. To do this, they needed funding. In the case of PBF, funding primarily came from bilateral donors (NORAD and UK’s Department for International Development), and the World Bank. These efforts were catalysed within the Health Results Innovation Trust Fund (HRITF). Individual leadership was critical to harness financial resources. For NORAD, a special advisor to the prime minister of Norway, who had previously worked at GAVI, had been “*very interested in looking further into the performance-based financing aspect*” of that institution, whose funding was based on performance. Soon after that, “*he got involved with the World Bank*” which was building a proposal on performance- and results-based financing, and NORAD came on board (I13_INTORG). All respondents referred to a single World Bank employee, a former Task Team Leader in an African country that pioneered the approach who had “*lobbied successfully for creating this HRITF within the Bank*” (I16a_INTORG).

#### Framing politically legitimate solutions to problem representations

First, DEs framed problem representations described above by strategically linking each constructed problem to popular and trendy solutions in global health and international development. Those matched PBF inherent principles (see Table 6), but they also came as responses to criticism arising from non-DEs (e.g. on the motivation debate). Initially, some actors were reluctant to embark on PBF; this reluctance decreased through the (re) emphasis of some problem representations, such as passive purchasing function (see point 1, Table 6). As such, PBF moved from passive to active purchasing. When the concept of “strategic purchasing” came into the debate in late 2016, PBF offered to operationalise that concept:

*Moving towards strategic… more strategic purchasing we think is associated with better… results. And… really the key thing that we’re looking for I think in this [strategic purchasing] agenda is how all the… energy and movement around… PBF can be used to trigger that, in the system.* (O4_International conference)

**Table 6:** Constructed problem with corresponding ideas and respondents’ quotes

Constructed problem	Popular concepts or paradigms to help solve the problem	Related quotes
1. Input-based financing systems with passive strategic function causing public service ineffectiveness and inefficiency	Renewed public management structures; strategic purchasing through output-based financing	*It is about public financing, it is a matter of giving back power to the state or more capacity to negotiate with the state […] It is about making the public system effective and I have this intimate personal conviction that public systems effectiveness is the best defense of their... sustainability: um, that ineffective public systems will eventually disappear.* (I17_TANSCO)
		*Still, I think [PBF] is a reflection on strategic purchasing issues, and it's still something important, and something positive for us... […] In the mechanism we say uh, here: the health service is supposed uh to provide such service, uh... at such a price, for such volume and uh... and he is paid according to what he actually does and not uh... something more random.* (I18_INTORG)
2a. Lack of accountability of public health spending	Output-based aid and better aid-tracking systems	*Donors tried several approaches: providing direct funding, relying on input-based financing that is, offering training, training people for... for inputs, uh... without really knowing whether these trainings are used, what is the use of all these books, what is the use of all these documents, et cetera… So we had... we were a little… about to falter, with a funding approach… that did not provide any result.* (I34_ACADINST)
2b. Lack of accountability of public health spending	Separation of (purchaser-provider) functions	*The implicit recommendation to African countries is that they can proceed towards universal coverage on the basis of the existing model: a national health service characterized by the State fulfilling all the roles: owner, employer, supplier, purchaser, regulator, administrator… A system in which health facilities are public administrations receiving their resources through line item budgets, often even “in kind”. It is precisely this status quo that PBF champions are challenging.* (I19)
		[NB: This quote is extracted from a blog entry posted by I19 and referred to by the key informant himself during the interview I19a_ACADINST]
3. Unmotivated and underperforming health workers	Setting (financial) incentives for health workers attached to performance indicators, and reinforcing supervision	*Of course, PBF brings staff motivation and we also often observe a change of behaviour on the part of the staff, who dev… almost develop a spirit of entrepreneurship; so they try to imagine strategies to receive more patients, and improve the quality of services […]. So there is a certain emulation, bringing in a spirit of entrepreneurship, and leadership, to attract more patients and to improve the quality of services.* (I55_NATGOV)
4. Highly centralised countries	Enhancing providers’ autonomy	*That’s autonomy for health centers at the primary level, […] instead of sending funding and controlling everything that practitioners can do with this money… even if in the cabinets there are far too many medicines of one kind, while it does not meet the needs. And then it's recognising the... value in the system of health providers, and allowing them to... […] feel valued.* (I25_INTORG)
5. Underperforming monitoring systems	Effective health information systems; data for decision making	*This is the first time that anybody has an information about… (chuckles) you know… whether it’d be the quality, the quantity, the financing of services… hum… and that is a whole kind of cultural (pause) change where people are actually understanding what’s going on. Hum… and that does contribute to making informed decision-making. Hum… we can track how well certain services are… hum… improving in terms of quantity and quality, where we couldn’t before.* (I08_INTORG)

PBF thus got linked to strategic purchasing, reportedly thanks to internal framing activities done by individual DEs within the World Health Organization. Strategic purchasing had (re) gained traction: organisations that had been reluctant were now eager to start supporting PBF. The international community saw introducing strategic purchasing as a systemic reform, and therefore it represented a more palatable idea.

Second, embracing concerns for country ownership and aid effectiveness, DEs framed PBF as a participatory, LMIC-driven strategy. This strategy included promoting a set of best practices which emerged from decades of PBF experimentations that each country needed to adapt to their own context(s). DEs thus crafted PBF as an adaptive reform yielding much support from LMIC governments. On the African continent, DEs specifically framed PBF as being led by African practitioners. Such framing enhanced legitimacy to PBF in SSA countries.

#### Shaping the ways to experiment PBF

Principally, donor money served to fund PBF pilot schemes across LMICs. As with most donor-funded programmes, it required strategies to ensure successful pilot scheme implementation, including setting rules of collaboration between actors. Non-DEs specifically discussed the operating rules of the World Bank/HRITF-funded pilot projects. They reported that a first strategy for these DEs was to design a standardised PBF model with a very structured set of guidelines for institutional collaboration and pilot project planning

According to an interviewee working for a NGO acting as DE, diffusing a PBF “blueprint” was important in order to make sure “*the power of the message*” would not be lost (I04_ PRIVNFP). Several interviewees noted a number of management, financial, and human resource constraints set by organisational DEs. In Table 7, we report on the specific testing package of World Bank/HRITF-funded schemes.

**Table 7:** Typical PBF pilot testing package, World Bank/HRITF-funded schemes

World Bank- and HRITF-funded schemes are typically implemented in the following stages: 1. Setting an independent project management unit at the central level 2. Drafting a PBF manual in collaboration with the Ministry of Health (i.e., operating procedures for contract agreements, including specifying PBF indicators and how rewards are calculated and distributed) 3. Arranging two competitive calls for tenders: 1) between private companies willing to act as purchasing agency or “contract development and verification agency”; 2) between private companies willing to act as fiduciary agency 4. Introducing a strict separation of functions through contracting the selected purchasing agency or “contract development and verification agency” 5. Training the multiple actors (i.e., regulator; purchaser; payer; healthcare providers) involved in the pilot scheme 6. Setting a steering committee at the government level and (possibly) a PBF unit within the Ministry of Health 7. Launching the pilot scheme in selected health districts 8. Ensuring smooth operationalization of pilot scheme (i.e., completion of contract agreements, verification, counter-verification, regular meetings of the steering committee) 9. Evaluating the pilot scheme (e.g., through a formal impact evaluation). *Source: Data aggregated from 57 interview transcripts and 10 observation notes*	

With such a high amount of donor control, informants highlighted issues of ownership that ought to be resolved. Implementers needed to involve government officials, to trigger the change of culture that PBF required, but that was often challenging:

*Consultants… do not sufficiently integrate people at the Ministry of Health, government officials. Even if they try to integrate them… some will continue to do business as usual.* (I25_INTORG)

Other interviewees expressed concerns about lack of government ownership. Concurrent strategies to secure ownership at ministerial level were developed when initiating pilot schemes. To achieve this, informants mentioned setting a PBF unit inside the ministry of health (MoH). Engaging the MoH was often considered insufficient. Interviewees representing organisational DEs emphasised the need to seek political dialogue directly with the ministry of finances rather than exclusively with the MoH.

According to DEs, government ownership also depended on identifying and promoting a “policy champion” who understood the complexities linked to PBF implementation and advocated for the policy solution. Sometimes, however, donors’ promotion of policy champions was controversial at country level because the donors’ choice was not deemed legitimate by governments. This discrepancy was considered a major hampering factor to successful pilot implementation. Complementary to that strategy was the provision of adequate and sustained technical assistance, i.e. coaching local champions and implementing teams. Several DE informants were mindful of their coaching role, arguing that it was a necessary step for local actors’ complete understanding of PBF.

#### Spurring emulation around PBF

Among LMICs piloting PBF, “flagship countries” like Rwanda served as a source of emulation. Indeed, according to DE informants, these countries successfully experimented and scaled-up PBF, and became success stories that inspired others. In many African countries, respondents noted that the Rwandan model was a source of inspiration for setting indicators and scheme architecture. An interviewee described a situation whereby an African country’s leader was very keen on scaling-up PBF at the national level, simply building on the Rwandan model and without piloting PBF at a smaller scale. Several interviewees, including DEs, worried about a somewhat “overconfidence” in the Rwanda model.

This emulation process was also driven by an organisational DE “*which operated as a travel agency*” (I40_PRIVNFP), as it funded and promoted multiple study tours to Rwanda and, later, to Burundi (which was also featured as a PBF “success story”) and other countries. Study tours (ranging from three days to a full week) were a key ingredient of policy emulation, and it usefully matched the framing of an Africa-driven policy. From the end of the 2000s, delegations – made of senior officials and sometimes health workers – from various SSA countries about to experiment pilot schemes traveled to other countries to get to know their respective PBF models. These delegations were typically enthusiastic when coming back to their home country. The study tours enabled them to “get a sense of reality” as to how PBF could be (successfully) implemented:

*It's really putting people in… situations where they can face concrete examples, in situations where they can… get inspired, mirror themselves!* (I46_INDCONS)

Despite such enthusiasm, other government officials voiced concerns about the need to contextualise the PBF model to country needs (e.g., in Cameroon and in Mali). The contribution of PBF study tours to policy diffusion was often alluded to with caution.

Besides study tours, DEs involved in the implementation of these schemes engaged in a number of peer-to-peer exchange gatherings, which also reinforced the African framing of PBF. DEs convened international gatherings whereby PBF implementers from African countries and other LMICs interact for a week. On these occasions, participants were encouraged to engage in socialisation activities and create connections – along the course of their PBF experience. Thus, there is a combination of genuine, spontaneous emulation (e.g., country teams communicating with each other on programme implementation components), and an explicit push by DEs to spur inter-country exchanges across the continent (e.g., a World Bank employee sending country teams to visit PBF units in other countries and “importing” Rwandan consultants to other countries) in order to subsequently copy certain features of a PBF scheme in some country and paste it in another country.

Study tour after study tour, gathering after gathering, the “community” of PBF experts expanded, building a group of “second phase-DEs”:

*You had a nucleus of people ten years ago that taught PBF and now you have… a sea of people who are experts and who are providing the support […]* (I53_INTORG)

Many informants indicated that this global community was leading PBF diffusion. Organisational DEs developed their own communities (thereby acting as network DEs): the *Results-Based Financing Health* community, managed by The World Bank and funded through the HRITF; and Cordaid’s *Multi-country PBF network*. An additional prominent network DE also spurred emulation among PBF practitioners: the *PBF Community of Practice*. Members of this network described a strong sense of belonging to this community.

Some non-DE respondents, however, questioned the idea of a genuine community of PBF implementers. They described the emulation processes as being artificially induced by external actors, and organisational DEs acting at the global level. Some non-DEs spoke of a certain pressure to go on study tours and attend international workshops. A respondent compared these strategies to a form of “*evangelism*”, by “*appeal [ing] to the feeling of belonging to a network*” (I51_ACADINST).

#### Controlling and disseminating PBF learning

Probably the most important strategy coined to favour PBF global diffusion was the development of an apparatus of learning strategies attached to PBF experimentation, which explicitly portrayed PBF as a “learning-by-doing” reform. This apparatus was developed by DEs – from initial training to results dissemination. It ensured a continuous control of the policy learning generated by the early PBF pilots. Different learning modalities were included: implementers and policymakers participated in PBF training sessions, accessed continuously updated implementation manuals, and were exposed to (scientific and experiential) knowledge on pilot schemes in various workshops, online fora, and publications.

**Fig. 1:**
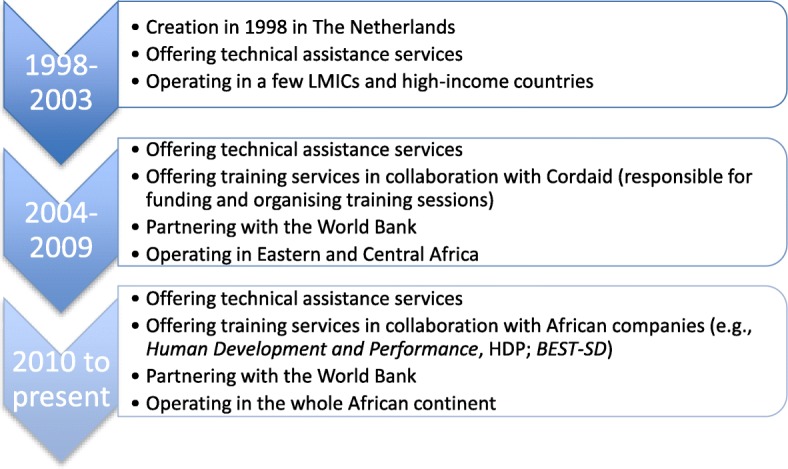
SinaHealth Company: a bit of history. Legend: *Source: I10a,b,c_PRIVFP*

The first major strategy of the learning apparatus was the development, funding, and promotion of international training sessions followed by cascade training at country level. For the World Bank, training represented a “*fundamental*” strategy to diffuse PBF, to “*ensure we had a critical mass… of people… in every country”* (I20a_INTORG). This method was done to facilitate country’s engagement in the approach. Providers of PBF training in SSA include public organisations, private companies, and not-for-profit organisations. The company *SinaHealth* was prominently featured in interviews (see Fig. 1 for details):

*And… I have to say [name of SinaHealth’s head removed] also has done a lot of trainings […], a lot of workshops: two-week drill down on PBF. A lot of policymakers passed through his classes. And… these are very dense courses […]. You know, like forty-five people in a place for twelve days and you hammer them and they walk out of it with a better understanding of what it is.* (I16a_INTORG)

This quote suggests very intense training sessions whereby trainees are presented with an undisputable policy solution (PBF). Trainers reportedly reviewed the theoretical underpinning and practical dimensions of PBF, emphasising a set of “*best practices*”. If 80% of these best practices got “*applied correctly*”, it was assumed that PBF would “*always work*” (I10a_PRIVFP). Independent of funding, almost all African pilot implementers received SinaHealth training: donors believed in the company’s capacity to train African nationals. Even projects not funded by the World Bank/HRITF got exposed to SinaHealth training sessions. Yet none of the interviewed DEs working at the global level seemed to have participated in a PBF training. They preferred to “*learn from the field*” and/or “*team up with people that know on how this works*” (I03_INTORG). Interviewees noted that after receiving training, trainees often joined the PBF Community of Practice (CoP) thereby building “fraternity” between SinaHealth and the CoP (I06_TANSCO). Therefore, training also reinforced emulation.

The second strategy involved the development of reference tools. As previously mentioned, organisational DEs developed their own PBF manuals detailing the specifics of their preferred PBF model. These practitioners’ guides were continuously updated to ensure incremental policy learning. Interviewees cited the World Bank’s PBF Toolkit in particular. However, some respondents expressed concern that it was used as a prescriptive tool because it was “*written by three advocates*” (I11_INTORG). Six respondents referred to it as “the PBF Bible”, and one raised concerns over “*following a recipe*… *without thinking*” (I51_ACADINST).

The third strategy, the “learning-by-doing” mantra, guided the funding and production of multiple types of knowledge on PBF experimentations. The most well-known type of knowledge was scientific impact evaluation. Building on Rwanda’s “*really famous*” study published in the *Lancet* (I02_INTORG), also considered the “*proof of concept*” for PBF (I19_ACADINST), the learning apparatus of the HRITF prominently featured impact evaluations using quasi-experimental designs. These preferred methods aligned with DEs’ economic-oriented representation systems. The two donors of the HRITF, building on long tradition in evaluation, explicitly requested this feature in view of building a “*global evidence base*” on PBF (I13_INTORG). The focus on impact evaluation at the HRITF motivated the development of parallel impact evaluations by other organisational DEs, such as USAID. Global DEs conceded that the results of these evaluations were not very impressive:

*The impact evaluations? They’re not – to be honest – they’re not that convincing, at least, there’s some strong elements… but they are not completely convincing.* (I13_INTORG)

At the World Bank, there was a certain tension between PBF advocates and researchers, the latter acknowledging that their organisation was “*obviously promoting PBF*” (I11_INTORG). These researchers reported pressure to generate positive results. DE respondents indicated that the reasons for mixed results owed to the methods chosen to evaluate the scheme, or to the variations in PBF scheme designs, i.e., if a PBF scheme was flawed, it could not yield positive results. Still, there was consensus among respondents that those mixed results should serve as lessons for improving PBF schemes. However, the mixed nature of the results seemed to have low visibility in SSA. After a presentation describing some of these results in a meeting taking place in an African city, one participant said:

*I do not understand these results you are presenting; if the World Bank promotes this strategy, it means it has been proven. After all, it’s an international institution that is behind it!* (O2_International conference)

The same positive bias emerged from several interviews with African respondents.

Before publication, preliminary results were typically shared by those who mandated the impact evaluations in the countries where the research has been done, using a “*participatory approach where… once some initial results… are put together, we kind of present this, [asking]: do you understand why, what’s causing these results?*” (I08_INTORG)*.* In spite of this approach, results uptake by policymakers was considered quite limited: respondents acknowledging that decisions to scale-up the PBF approach were often decided before research results were made available, or in spite of them. According to an interviewee, this could be explained by a mismatch between government’s interests – looking for policy relevance – and academics wishing to “*publish some nice papers*” (I05_INTORG). Outside the World Bank/HRITF, criticism arouse from many informants, including several individual DEs, who pointed to the risks of overlooking non-quantifiable effects of PBF. Several DEs linked to the main organisation reasserted the need to expand rigorous evidence, while acknowledging that it was also important to account for operational and qualitative data. According to DEs, this aspect got integrated in HRITF-funded impact evaluations from 2013 onwards, which sometimes included a qualitative component.

Impact evaluation results were shared with implementers of PBF pilot schemes and policymakers in international workshops and conferences. Respondents specifically referred to the World Bank/HRITF’s learning and knowledge exchange workshops. With time, these yearly workshops became opportune for sharing another type of knowledge, i.e. experiential knowledge on PBF. Hence workshops also included peer-to-peer exchanges between PBF pracititioners. While some DEs promoted the image of participatory workshops (e.g., giving voice to practitioners), other DEs suggested that the World Bank was deliberately controlling the content disseminated in these workshops, thus allowing little space for alternative views:

*Over time there’s been a bit more opening to other organisations to participate [in these workshops] but… it has been really a Bank-centered thing, from the start. I think the concern is there are already so many […] people involved, that everybody feels a bit dissatisfied… that they didn’t get exactly what they wanted… out of it. So by opening up even more, maybe… they won’t be able to control at all what… […] what is discussed during these [workshops].* (I08_INTORG _211116)

According to interviewees, the PBF CoP developed more horizontal (and possibly less controllable) forms of knowledge, building on experience and not only academic studies. CoP facilitators searched for participatory formats to disseminate knowledge, notably through developing an online forum and blog entries on implementation challenges, and organising face-to-face workshops:

*In fact, the CoP gives meaning, it gives… a validation… in the sense: “I'm not alone in doing what I'm doing”. […] And in those workshops, you try to create collective enthusiasm.* (I19a_ACADINST)

The CoP’s strategies emphasised the feeling of emulation: learning processes reinforcing emulation.

## Discussion

Our study analyses the problem representations (re)shaped and spread by PBF diffusion entrepreneurs at the global level, and how these representations reflected DEs’ own belief systems and interests, often in contrast with non-DEs’ representations. Our study examines the specific strategies DEs engaged in to effectively diffuse PBF, through framing the PBF discourse and inducing certain forms of policy experimentation, emulation, and learning.

Results from our empirical analysis concur with our first and second propositions formulated on the basis of a literature review on PBF, namely “the merging of resources and types of authority” and “the strategic use of policy frames” increase the likelihood for a policy innovation to diffuse [1]. This analysis is congruent with previous empirical studies on PBF: Barnes *et al.* argued that these actors have combined a wide range of resources and implemented strategies to shape the direction of the diffusion [29]. Yet, the present study revealed that a key ingredient was necessary to achieve this: long-established trust between DEs. Existing relationships between DEs enabled them to rely on each other’s resources and authority, and pool their efforts towards their common project – diffusing PBF. This postulation is consistent with multiple studies on the diffusion of information in international development, which feature the prominent role of social relationships in leveraging knowledge into action [30].

Barnes *et al.* described PBF DEs’ representations systems and motivations as homogenous [29]. While we found similar representation systems (around economics), there were differences across individual DEs in favoured economics schools of thoughts. Notably, not all of them advocated for healthcare providers’ competition. However, DEs did agree on certain fundamentals – the usefulness of bringing in economic concepts to public health provision. This was a key facilitating factor for DEs. In particular, as featured in previous works [31–33], the economic-oriented language reflects the World Bank’s continued inclination to view healthcare provision as a “market commodity” rather than a public good [34]. This inclination is reflected in their coframing (together with the Rockefeller Foundation) of the “investment case” for UHC, which has been endorsed by prominent economists [35]. Many global actors have bought into the idea that investing in health yields economic returns – an idea pushed forward by the WHO Commission on Macroeconomics and Health [36].

The economic culture was shared by many DEs, and it critically oriented their views about the ways global development could be achieved. Works on the diffusion of new public management (NPM) drew similar conclusions [37–39]. NPM represented a policy innovation that got diffused by external actors in many LMICs by mobilising an apparatus of strategies. Like NPM, PBF offers a typical example “where there is general agreement on the cause and effects of managerial techniques by a community of ‘experts’” [38]. In fact, communication theory contends that it is easier for policy advocates to communicate with people who share “similar frames of reference” [40].

The rhetoric strategies developed by both DEs and non-DEs showed that discourse is a process by which actors provide “interpretive frameworks that give definition to [their] values and preferences and thus make political interests actionable” [41]. This process entailed simplistic subjectifications as well as overlooking critical issues on both sides. We also found that DEs’ interests were not only political (i.e., gaining visibility and/or boosting career and reputation on the global arena), but also financial (i.e., pursuing donors’ favoured output-based aid “trend”). Pursuing donors’ interest in measuring aid effects also aligns with what we could call a generalised trend towards “quantifying results”. The latter is also reflected in the broader *Sustainable Development Goals’* agenda with its focus on metrics and innovative financing mechanisms. This trend illustrates the spread of both a “performance ideology” and audit culture that previous studies have identified [42, 43]. PBF matched this global push for performance, by featuring effectiveness and accountability as the key to solve the represented problems. These keywords found a particular echo across SSA for two interrelated reasons: 1) the relative hopelessness that the health status of this specific region was eliciting among the international community; 2) the need for bold, transformative policy ideas that could reverse the tendency (and achieve results similar to those of other regions). PBF three key features gave hope for promising results: an output-based approach breaking away from business-as-usual funding systems; new forms of financial incentives to increase health workers’ motivation; enhanced autonomy at facility- and decentralised-levels.

Our empirical proposition postulating that “increasingly polycentric governance arrangements foster the diffusion of policy innovations” [1] is relevant. Indeed, the PBF global community – DEs – included transnational networks as well as many individuals (experts and consultants) who exercised crucial governing power. This level of involvement was made possible by the polycentric nature of global health governance, which enables the participation of a wider range of actors to policymaking. These results are consistent with Lee & Goodman who highlighted that the health financing global elite had “come to dominate policy discussions through their control of financial resources and […] control of the terms of the debate through expert knowledge, support of research, and occupation of key nodes in the global policy network” [10]. On the diffusion of NPM, Common identified the same pattern [38]. The specificity of PBF is that the rapid expansion of this transnational community yielded a generation of individuals, i.e. Africa-based experts who bought into the PBF solution and diffused it across the continent, hence becoming “2^nd^ phase DEs”.

Our study concurs with others that the discourses produced by powerful and highly-motivated global actors have legitimising effects on the policy they intend to promote [44, 45]. Indeed, DEs appeared to be successful because they had influence on the global arena (based on their reputation) and the financial authority to spread such discourses. Consistent with Cairney’s recent work on policy entrepreneurs, “our” DEs achieved this “through using persuasive stories […] combining facts with values and emotional appeals (with heroes and morals), engaging in coalitions and networks to establish trust in the messenger” [46]. Heroes were health providers or populations, and morals revolved around blaming the system not conducive of quality services provision (i.e., with health workers trapped inside the can-do will-do gap).

Coalitions and networks were made up of those transnational communities that developed both informally (e.g., among international experts) and formally (e.g., the CoP), as the result of deliberate strategies initiated by the above-mentioned global elite. These communities benefitted from resources and authorities (based on the reputation and expertise of high-profile individual and organisational DEs) that enhanced their credibility in the global arena. Did these transnational communities also enjoy legitimacy to exert power based on inclusive and transparent processes (i.e., “input legitimacy” [47])? Their transparency was not optimal (e.g., debates often silencing key issues), but improved with time (e.g., acknowledgment of mixed results yielded by PBF pilots). As for inclusiveness, even if most individual and organisational DEs came from high-income countries, the strong participation of African practitioners in these communities appears to have mitigated concerns for the lack of voices from LMICs. However, the representativeness of “ordinary people” by these African practitioners can be questioned. This feature needs to be further explored by future empirical research, also because the above-mentioned global elite has been largely shaping the debate.

Our findings showed that the idea of introducing individual incentives leads to the production of subjects (e.g., entrepreneurs), as Bacchi would say. Referring to a 2001 study on students’ repayment of their loans, she asserts that “the practice of repayment based on salaries” renders students – now appealed to financial gain – “governable” [48]. A similar reasoning could apply to the practice of providing financial awards based on performance – it could be simply an attempt to render them more governable. We also identified a number of representations silenced in the PBF policy as promoted by DEs: equity was seldom mentioned, as well as health providers’ accountability to SSA populations (as opposed to accountability to donors or government authorities). This concurs with available literature on PBF, which explicitly calls for accounting for equity when designing and evaluating PBF projects [49], and for questioning the effectiveness of PBF community verification procedures [50].

Pilot programmes of PBF in SSA are promoted, designed, funded, implemented, and evaluated by international institutions [51]. PBF pilot packages were primarily promoted by these actors, with local implementers enjoying limited ability to manipulate and/or control the intervention. This factor caused multiple ownership concerns, as previous studies also have shown [51, 52]. This feature is, however, not specific to PBF – this issue has been observed in many donor-driven pilot programmes. What made experimenting PBF so distinct was the sustained in-country coaching and technical assistance provided by foreign individuals, and the heavy reliance on policy champions who could understand the complexities of PBF and successfully advocate it in their respective countries. The Cameroon case is salient [53].

The HRITF’s idea of looking at the effects PBF generates, while financially investing in this reform, can be initially lauded. However, the narrow focus on quantitative impact evaluations, and the potential tension between advocates and researchers employed by the organisation which mandated these evaluations are problematic [54]. These features could suggest an intention to control the discourse on PBF. In addition, the large investments in impact evaluations have yielded mixed results. These results are consistent with results presented in published systematic reviews [2–4]. Interestingly, while the mixed nature of results was acknowledged by global DEs, informants from SSA frequently demonstrated a positive bias towards the impact of PBF. The discrepancy is most likely owed to barriers in access to information. This gap might also reflect a social desirability bias; perhaps those respondents had no interest in admitting that results were mostly mixed, possibly due to the fact that their job depended on the continuation of PBF in their respective countries. More generally speaking, the political economy of development aid projects, whereby national actors embrace donors’ projects to secure additional funding for their respective countries [51], may lead SSA policymakers to adopt a cautious political correctness *vis-à-vis* PBF. This pattern has been demonstrated in a seminal book about national actors’ perceptions of donor-driven development projects in Sahel [55]. In addition, there was a low uptake of PBF research results by SSA policymakers. Congruent with Schneider [56], respondents conceded that results from scientific evaluations were not used by policymakers to inform their decisions regarding the alteration, scale-up, or stopping of PBF. This concession concurs with the lack of political consideration of results even when emerging from clinical studies, such as those conducted on pre-exposure prophylaxis to prevent HIV infections. It was often implemented in many countries including in SSA [57] despite inconclusive evidence [58]. Similar patterns were observed about the national roll-up of *Revenu de Solidarité Active* in France [59]. These comparisons suggest that policy is often indifferent to scientific evidence [60].

In addition to producing impact evaluations, DEs organised international gatherings, study tours, and training contents that diffused tacit knowledge on PBF. Rather than actual policy learning (i.e., “information or experience from other units for better-informed policymaking” [1]), such activities achieved more policy emulation, whereby event participants developed, through sharing this tacit knowledge, a common interest to implement PBF, and a depoliticised, primarily technical language about PBF. Indeed, countries were prompted by donors to reproduce features of schemes inspired from elsewhere. Similar patterns have been observed on the diffusion of health microinsurance: recognised global institutions such as the International Labour Organization (ILO) and Micro Insurance Academy publicised a variety of handbooks inspired from reputable success stories, which described in great detail how to develop and implement “standard” insurance schemes in LMICs [61, 62]. These organisations also supported study tours to countries that had insurance success stories (e.g., RSBY in India, and Asmade in Burkina Faso). ILO even developed a guide for organising study tours [63]. All of these elements bring up critical issues about the role of externally driven, “constructed” learning apparatus officially aiming at evidence-informed policymaking, and call for further investigation.

Applying Bacchi’s approach to the PBF case was both enriching and challenging. On the one hand, her dimensions on the subjectification produced by discourse, and omissions in problem representations, were particularly useful and relevant to our empirical results. On the other hand, we faced a challenge: Bacchi shifted from policy representations to problem representations analysis, which entailed quite a different standpoint. We described the shaping and ensuing promotion of problem representations. We also demonstrated how DEs strategically framed problem representations as opportunely and comprehensively addressed by PBF and its core principles. However, our analysis of DEs’ other strategies emphasised representations directly related to PBF, because we aimed at unraveling DEs’ controlling of policy experimentation, emulation, and learning. Besides, applying Bacchi to interview data, which featured contrast between two groups (DEs and non-DEs) considered as a separate set of respondents/representations involved somehow departing from Bacchi’s original intention: Bacchi’s framework usually applies to textual data, irrespective of who produces them. However, given the nature of the current debate on PBF, which features a high polarisation [64], it was relevant to analyse the contrasts across representations on both sides.

Like Bacchi, Naudet suggests that policy solutions typically precede problem definition in SSA contexts [55]. Naudet showed that donors’ (development or disbursement) objectives, and the political instruments at hand, often determine needs’ assessment (and thus problems identification). In the case of PBF, we observed that DEs deliberately implemented strategies to emphasise certain problem representations in ways that were politically opportune (e.g., input-based systems fatigue), and linked PBF to popular frames (e.g., country ownership) so as to (re) assert the legitimacy of their policy solution in a contested landscape. Our study thus reveals a more complex pattern than that of Naudet’s, in which the policy solution constantly evolves and adapts to align with the broader development debates occurring on the global arena. The opportunistic linking of PBF and strategic purchasing was a case in point, whereby DEs succeeded in reframing PBF in a way that attracted more buy-in within the global health arena.

For the sociologist of science Callon, “*translation*” is the dynamic process by which actors initially different, end up (by negotiation or conviction) entering into a dialogue around a common representation of a problem [65]. Callon suggests that solution promoters, who engage in problematisation like DEs, seek actors who may have an interest in getting on the case, and try to convince them that the promoted problem representations make sense. To achieve this, promoters develop “a set of actions by which a group of promoters strives to impose and stabilise the identity of the other actors that it has defined by its problematisation”, i.e. “*interessement*” [66]. Interessement is based on a certain assumption of what the actors are, want to engage, and associate with: this involves establishing relationships with them. Thus, in the same fashion PBF DEs develop strategies, promoters diffuse their problem representations through multiplying social interactions: meetings, etc. On these occasions, promoters continuously have to negotiate, persuade, and reformulate their argument to adapt to potentially interested actors, who are themselves in constant evolution [65]. When the interessement scheme succeeds, problematisation gets validated. This outcome is precisely what happened when PBF got reframed as a key strategy to switch from passive to (active) strategic purchasing.

Our study employed a strong theoretically-informed approach to analyse a rich corpus of qualitative data. Repeated interactions with interviewees (cross-checking information through additional interviews, and letting participants comment on the study’s preliminary results) enhanced our results’ credibility. The detailed description of the methods used to collect and analyse data provides a strong dependability. We ensured transferability through a detailed description of participants’ characteristics: other researchers may use similar inclusion criteria in order to reproduce this research in other settings and on another topic related to international development. In short, our results’ confirmability is high [67].

The main limitation of this study is that respondents from international organisations (in particular, one organisation: the World Bank) dominate the sampling. While this one-sidedness suggests that they represent the most obvious promoters of PBF, non-profit organisations also played a crucial role in PBF global diffusion. Three of our respondents had moved from NGOs to international organisations at the time we analysed their interview transcripts.

## Conclusion

Bacchi’s poststructural approach proved useful to analyse the construction of global health problem representations and the strategies set by global diffusion entrepreneurs to spread these representations and shape the discourse. The diffusion of PBF has benefited from problem representations and policy frames that were cleverly and strategically implemented by global diffusion entrepreneurs, sometimes in reaction to criticism expressed by other academics and international organisations. Future research is needed to further analyse the role played by global diffusion entrepreneurs in creating and promoting “2^nd^ phase DEs”, i.e. African experts who diffused the PBF solution across the African continent and at country level. Further assessment of the policy indifference to research results in Africa is also critical. Other authors ought to study additional global development policies to investigate how problem representations, motivations, resources, and strategies of actors are used to shape the global discourse and influence policy diffusion.

## Additional file

Additional file 1: Interview guide. (DOCX 160 kb)

## Abbreviations

DE(s): Diffusion entrepreneur(s)
GAVI: Global Alliance for Vaccines and Immunization
HRITF: Health Results Innovation Trust Fund
ILO: International Labour Organization
LMIC(s): Low- and middle-income country(ies)
MoH: Ministry of health
NGO(s): Non-governmental organisation(s)
NORAD: Norwegian Agency for Development Cooperation
NPM: New public management
PBF: Performance-based financing
SSA: Sub-Saharan Africa
UHC: Universal Health Coverage
WHO: World Health Organization
WPR: What’s the problem represented to be
