# Supplementary material for: How is the discourse of performance-based financing shaped at the global level? A poststructural analysis
Source: Global Health. 2019 Jan 15;15:6. doi: 10.1186/s12992-018-0443-9 (PMC6332594; doi:10.1186/s12992-018-0443-9)
Supplement: Supplementary file 1 — Interview guide. (DOCX 160 kb) [file 12992_2018_443_MOESM1_ESM.docx]

**Interview guide**

**Analysing the diffusion of performance-based financing**

| **Archival Number** |  | | **Name of Interviewee** | |  | | |
| --- | --- | --- | --- | --- | --- | --- | --- |
| **Position & Affiliation of Interviewee** | | |  | | | | |
| **Name of Interviewer** | | |  | | | | |
| **Mode of Interview** | | | Face to face Phone | | | | |
| **Audio recorded** | | | Yes No | | | | |
| **Date** | | | **DD** | **MM** | | **YYYY** | |
| **Start Time** |  |  | **End Time** | | |  |  |

Script: *I am Lara Gautier, a PhD student at Université de Montreal in Canada and Université Paris-Diderot in France. The aim of this research project is to learn about the processes that led to PBF policymaking. This study is part of a larger research evaluation program on “Results-based financing for equitable access to maternal and child health care in Mali and Burkina Faso”. This program is run by a consortium of Mali- and Burkina Faso-based research NGOs and researchers from the University of Montreal^[[1]](#footnote-1)^.*

*I have asked to interview you, because you are a key person with knowledge and insight regarding the emergence of PBF as a global health policy and I am interested in learning your opinions and personal experience regarding the PBF policy.*

*I have reviewed the procedures for the interview during the consent process. Do you have any questions before we begin?*

| **Question** | | | **Prompts** | | | **Comments** |
| --- | --- | --- | --- | --- | --- | --- |
| 1. **Could you tell me a little bit about your background?** | | *How did you come to your current position? What was your personal trajectory?* | | | | |
| 1. **How long have you been working in this institution?** | | | | | | |
| 1. **How do you feel about your job?** | *What would be the most fulfilling or exciting, or the most challenging or frustrating in your job?* | | | | | |
| 1. **How did you first hear of performance-based financing (PBF)?** | | | | | | |
| **A. SECTION ON REPRESENTATION SYSTEMS, MOTIVATIONS AND RESOURCES** | | | | | | |
| 1. **For you, what does this global policy represent? How would you define it?** | | | | | | |
| 1. **Could you tell me a little bit about the ideas and values that are behind the development of PBF in LMICs?** | | | *What is the purpose of the PBF policy? What policy issues does it address?* | | | |
| 1. **According to you, what are the possible reasons for choosing to implement this policy in many LMICs, particularly in African countries?** | | | | | | |
| 1. **What do you think about the effect(s) of this policy?** | | | | | *Do you believe that this policy works well in LMICs? Why or why not?* | |
| 1. **According to you, what helped the most in shaping the development of this policy?** | | | | *What impact did this have? How much help were these facilitators?*  Facilitators might relate to:   - Individual skills/knowledge - Positive evaluation - Political factors - Policy/program topic factors | | |

| **B. SECTION ON STRATEGIES** | |  |
| --- | --- | --- |
| 1. **What types of documents are used about PBF and why do you think they are relevant resources?** | - *Primary research studies; such as impact evaluations published in academic journals* - *Secondary research articles (reviews) such as systematic reviews or research summaries* - *Government reports or other unpublished (grey) literature, including evaluations of policies or programs from other organisations* |  |
| 1. **Would you consider that evidence (any type of knowledge resources) informed the development of the PBF policy in any way? How did they?** | *What is your assessment of the importance of the contribution of knowledge resources to the development of the PBF policy overall?* |  |
| 1. **For instance, can you name a specific knowledge resource on PBF used in your organisation?** | *Is it commonly referred to in your organisation?* |  |
| 1. **Can you describe the ways these resources were used in the development of PBF in countries? What do you think about these?** | *Can you describe how research was used? Were there concerns about the use of generalised research for instance?*  NB: Generalised research might result in overconfidence in the benefit of particular initiatives |  |

| 1. **Could you tell me about the consultation process with experts, reference groups or researchers to inform this policy?** | | |  |
| --- | --- | --- | --- |
| 1. **Did you play a specific role in the development and/or diffusion of this global policy? If yes, what was this role about? Who did you work with?** | | NB: Understanding the role of the interviewee is very important to the validity of the interview. Was their role central, marginal, advisory? Did they produce/contribute to/direct some documents on PBF? | |
| 1. **As you may know, there is a wide range of activities for governments’ representatives and local experts to familiarise with PBF. Do you know what these activities are? Can you describe them?** | | *What are their objectives? To what extent do you think they contribute to policymaking in LMICs countries according to you? Could you give some examples?* | |
| 1. **Have you contributed to organise one of these activities? If you have, how are government representatives selected to participate in these activities?** | | | |
| 1. **Could you please describe for me how these activities (e.g., workshop or study tours) take place and what their objectives may be? You may give a particular example if you wish.** | | | |
| 1. **In your opinion, how do training or study tours help convince national decision makers?** | | | |
| 1. **If not, have you organized or attended at least one special event on PBF, e.g. a conference session on PBF?** | | | |
| 1. **Do you follow the ongoing discussions on PBF, such as through the PBF Community of Practice or webconferences on PBF?** | *If yes, what do you think about it?*  *If no, why not?*  *To what extent these discussions inform countries decision makers according to you?* | | |
| 1. **In your opinion, what has been the response by donors and local governments to PBF?** | | | |
| 1. **Who else do you think it is important that we speak to (if more than one person nominated, ask interviewee to rank in order of relevance/ importance)?** | | | |
| 1. **Are there any resources or documents you would recommend we consider?** | | | |
| 1. **Is there anything you think has been missed or that you think is important for us to be aware of?** | | | |

1. This project is part of the Innovating for Maternal and Child Health in Africa (IMCHA) Initiative, a seven year, $CA36 million multi-donor partnership funded by Global Affairs Canada, the Canadian Institutes of Health Research, and Canada's International Development Research Centre [↑](#footnote-ref-1)
